# Co-creators, not bystanders: Advancing meaningful youth engagement in antimicrobial resistance response in Africa

**DOI:** 10.1371/journal.pgph.0005405

**Published:** 2025-12-01

**Authors:** Daniel Waruingi, Yusuf Babatunde, Hafeez Hamza, Gathai Mundia, Mayowa Akinpelu, Niniola Williams

**Affiliations:** 1 Department of Research, Zihi Institute, Nairobi, Kenya; 2 African Youth Antimicrobial Resistance Alliance (AYARA) Task Force, Lusaka, Zambia; 3 Alliance Against Antimicrobial Resistance, Ibadan, Nigeria; 4 Ducit Blue Foundation, Abuja, Nigeria; 5 Africa Public Health Student Network Initiative (AfricaPHSN), Abuja, Nigeria; 6 Dr. Ameyo Stella Adadevoh (DRASA) Health Trust, Abuja, Nigeria; University of Oslo Faculty of Medicine: Universitetet i Oslo Det medisinske fakultet, NORWAY

## Abstract

Antimicrobial resistance (AMR) is a critical and escalating global health threat, with sub-Saharan Africa bearing one of the highest burdens. Despite young people comprising nearly 70% of the region’s population, their involvement in AMR decision-making remains minimal, often limited to tokenistic roles or awareness campaigns. This essay calls for recognizing youth as co-creators and strategic partners in AMR mitigation. We analyze the current state of youth engagement in AMR initiatives across Africa, highlighting systemic barriers such as inadequate access to resources, structural exclusion, and entrenched power imbalances. Through examples of successful youth-led programs, we demonstrate how youth-driven innovation, advocacy, and community mobilization have generated sustainable impact. Building on the WHO’s Global Consensus on Meaningful Adolescent and Youth Engagement, we propose a practical framework to institutionalize youth participation in AMR initiatives. This framework emphasizes structural integration, intergenerational collaboration, equitable access to funding, capacity-building, and robust accountability mechanisms. We argue that meaningful youth engagement is both a moral imperative and a strategic necessity to achieve sustainable, inclusive, and context-sensitive solutions to AMR in Africa.

## Introduction

Antimicrobial resistance (AMR) is one of the biggest global health problems that is facing the world today [1–3]. AMR occurs when microorganisms such as bacteria, viruses, fungi, protozoa, and helminths develop traits to withstand the effects of antimicrobials, making them ineffective - as a result, infections become harder to treat [4]. By 2050, AMR is estimated to cause 8.22 (6.85–9.65) million associated deaths and 1.91 (1.56–2.26) million attributable deaths [5]. AMR affects the attainment of Sustainable Development Goals [6,7] and if not effectively addressed by 2035, AMR is projected to cost the global economy $443 billion annually in lost workforce productivity and $412 billion per year in additional healthcare costs [8].

The highest burden of AMR is in Sub-Saharan Africa, with an estimated 1.05million deaths associated with bacterial AMR and 250,000 deaths attributable to bacterial AMR in 2019 [9]. The high bacterial AMR burdens result from both widespread resistance and the high incidence of critical infections like respiratory, bloodstream, and intra-abdominal infections [10]. The other driving factors of AMR include limited lab infrastructure for proper diagnosis, lack of regulation leading to antibiotic misuse, insufficient access to second- and third-line treatments, poor sanitation and hygiene, counterfeit or substandard drugs, and a general lack of awareness on AMR [11–18].

It is important to recognize that the age bracket for “youth” varies across different countries and international organizations. While various entities such as United Nations (UN) define youth differently, this paper adopts the African Union (AU) definition of youth, which includes individuals aged 15–35 years, as outlined in the African Youth Charter. This definition aligns with the regional demographic and social context of Africa as well as the focus of these commentary [19]. We urge you to interpret the paper through your understanding of youth and age bracket as per your context.

The demographic profile of Africa is evolving, marked by a swiftly expanding youth population poised to drive social, economic, and political advancements. In numbers, nearly 70% of sub-Saharan Africa’s population is under 30 years old [20] and young people constitute a substantial segment of the healthcare workforce, playing a crucial role in shaping the region’s future. International human rights law safeguards the right of young people to participate in decisions, institutions, and processes that impact their lives. The United Nations General Assembly (UNGA) resolution A/HRC/RES/35/14 urges all states to promote and ensure the full realization of human rights and fundamental freedoms for youth, empowering them to actively contribute to the political, civil, economic, social, and cultural development of their countries [6,20]. In essence, meaningful youth engagement ensures that youth are valued partners in shaping programs, policies, and decisions that affect their lives, now and in the future.

Youth are increasingly recognized as drivers of innovation, advocacy, and social change [19]. However, AMR strategies rarely incorporate youth voices, insights, and solutions into decision-making frameworks. The young age profile and youth exclusion in program design undermine the effectiveness of current intervention strategies.[21]. The absence of systematic meaningful youth engagement in AMR policy and implementation processes limits the effectiveness of interventions, a critical gap that could be addressed by in leveraging the potential of this demographic. Additionally, unconscious biases and misconceptions about young people’s capabilities often limit their inclusion in critical AMR efforts. These biases result in tokenistic involvement, sidelining the creativity and perspectives of youth. This commentary developed by young authors who have extensive experience in youth engagement will serve as a practical framework and address these biases by providing clear and actionable steps for meaningfully engaging youth as co-creators in AMR mitigation. This is expected to trigger more robust youth engagement and participation in AMR interventions.

This commentary argues the prioritisation of youth engagement policies and implementation frameworks in the fight against AMR. It argues that the inclusion of young people in decision-making processes is not only a moral imperative but also a practical necessity to ensure an inclusive, sustainable and equitable AMR response. Youth should be recognized as co-creators and important stakeholders in AMR mitigation efforts. There is an urgent need for policies that prioritize meaningful youth engagement for young people to contribute their energy, creativity, and knowledge in AMR mitigation efforts.

### Current landscape of youth engagement in AMR initiatives

Although the current landscape of youth engagement in AMR initiatives across Africa illustrates efforts to include young people in AMR mitigation, meaningful and sustained engagement is limited. Exclusion of young people in important discussions risks producing research outputs, making policies, and recommending practices that misunderstand their interests. As such, the intervention are often ineffective reflecting the misalignment of particular needs, realities and priorities [22].

Youth are key stakeholders for health policies and actions, but they encounter critical underrepresentation in decision-making processes and platforms. Numerous youth-led non-profits and civil society organizations across Africa have been actively working towards mitigating AMR through creating awareness, policy and advocacy, research, among other interventions. However, there is a lack of comprehensive engagement, specifically in areas such as program design, policy development, and practical implementation [22].

While there are few organizations and international initiatives recognizing the role played by young people in AMR mitigation, most of them believe that youth are best suited to create awareness only. Further, most of the existing initiatives are either tokenistic or ad hoc, with youth engagement not entirely drafted into the program monitoring, evaluation or organizational strategic plan of the particular organizations. A scoping review conducted in 2021 on adolescent participation in health research documented widespread tokenism and limited decision making for youth participants [23]. An umbrella review similarly concluded that across health research programs, youth engagement is often episodic and poorly embedded in governance, monitoring, or evaluation systems [24]. As such, there is a need for more structured and formalized frameworks geared towards assessing and awarding roles to youth based on their actual capacities rather than perceived capabilities. Such frameworks should ensure that youth participation translates to long-term and actionable outcomes rather than immediate output.

Empirical studies from LMIC settings show that youth engagement can shift antimicrobial-use behaviors and expand stewardship. For example, in Nepal, a qualitative evaluation found youth influenced parental antibiotic behaviors and community discourse when engaged through school-based AMR activities. In Tanzania, peer-reviewed reports of AMR School Clubs describe 11,552 students reached across 25 schools, with youth acting as community “antibiotic guardians” and propagating hygiene and rational-use beyond schools [25]. [26]. Youth-led and youth-serving organizations on the African continent such as Students Against Superbugs Africa (SAS Africa), Africa Public Health Student Network Initiative (AfricaPHSN), African Youth Antimicrobial Resistance Alliance Task Force (AYARA-TF), Ducit Blue Foundation, AMR Now, One Health Society (OHS), and the International Pharmaceutical Students Federation African Regional Office have shown the impact of youth-led initiatives in the fight against AMR and many CSOs in Africa and beyond have spearheaded work to develop youth AMR champions.

SAS Africa’s AMR Ambassadors Program for Young People in Africa project which was implemented in 14 countries, led to increased knowledge and interest in AMR among 30,000 youth between June 2022 and January 2023 [27]. Many clubs and NGOs were formed from the project with several initiatives aimed at curbing AMR such as AWARE Ghana in Ghana; AMR Academy at Minna University (Nigeria); development of an Educational Handbook for primary school pupils in Tanzania; AMR clubs at the University of Ghana (Ghana), Usmanu Danfodiyo University (Nigeria), and Makerere University (Uganda); and the development of a sustainable framework on mainstream AMR media engagement in Cameroon [27]. The Ducit Blue Foundation One Health AMR Pan-African Internship/Mentorship Programme is a succession planning initiative that engages youth in AMR mitigation while nurturing them as future public health leaders. Over three cohorts, the programme has supported 51 youth from 13 African countries, equipping them with the skills to drive impactful change [28]. Youth alumni from the program have gone on to cascade knowledge to over 5,000 people in the communities and established NGOs such as the Alliance Against Antimicrobial Resistance (Nigeria), AMR Intervarsity Training Programme (Nigeria). Further, 3 youth alumni have received global recognition, including the prestigious Diana Award, highlighting the transformative impact of empowering youth to lead AMR solutions across Africa [29]. Similarly, African Youth Antimicrobial Resistance Alliance Task Force (AYARA-TF) is a consortium of 14 youth-led organizations in Africa formed under the guidance of ReAct Africa - one of the few international organizations recognizing the efforts of youth - and is set to shape the fight against AMR across Africa through concerted collaborative efforts among members [21]. The first phase of the One Health Development Initiative African Youths for AMR Communication Project focused on increasing the ability of youth to implement risk communication and community engagement initiatives for AMR. As a result, 41 youth champions from Nigeria, Cameroon, Rwanda, Tanzania, and Malaysia were trained on AMR risk communication and policy advocacy and then went on to further train 362 youth volunteers [30]. Collectively, these empowered youth reached over 7.6 million persons on AMR via various activities during the World AMR Awareness Week 2024 [30]. This multiplier effect of youth engagement for AMR was also evident in the Dr Ameyo Stella Adadevoh (DRASA) Health Trust’s AMR school program in Nigeria, where through the establishment of Health and Hygiene Clubs in secondary schools, 891 students in 30 schools were trained over one academic term to become champions of AMR in their schools, homes, and communities. These students, who demonstrated a 161% increase in knowledge score, went on to reach 8,323 persons within their circles of influence and implemented 1,366 independent AMR activities outside their school Club [28].

The results and impact of youth involvement in AMR response efforts are clear, yet despite these examples, there are barriers that youth face in engaging with AMR initiatives. This includes lack of access to information, resources, and platforms for participation. The lack of access to information and resources is one of the biggest challenges for youth engagement in AMR. In fact, from AMR educational initiatives in Malaysia that show the impact when implemented among adolescents [7] to Tanzanian youth who are equipped as AMR champions and go on to multiply the impact within their communities [31] there is evidence that providing youth with the information and resources they need to act can result in significant youth contributions to the fight against AMR.

### The case for meaningful youth engagement in AMR efforts

Meaningful youth engagement has different definitions to different people due to diversity of backgrounds and context. According to Women Deliver, meaningful entails inclusion of young people in all stages of developing, implementing, monitoring and evaluating programs, policies and investment of resources. According to the WHO’s Global Consensus Statement on Meaningful Adolescent and Youth Engagement, meaningful youth engagement is described, as “*intentional, mutually-respectful partnership between youths and adults where power is shared, respective contributions are valued, and young people’s ideas, perspectives, skills, and strengths are integrated into the design and delivery of programs, strategies, policies, funding mechanisms and organizations that affect their lives, and their communities, countries and world.”* [32]. This paper will utilize the definition by the Global Consensus Statement on Meaningful Adolescent and Youth Engagement by the World Health Organization which has been adopted by many institutions.

At the center of all definitions of meaningful youth engagement are principles intended to ensure equity, autonomy, recognize intersectionality, and the desire to quash exploitative, extractive, and disingenuous engagement of youth. It is important to acknowledge meaningful youth engagement as a participatory process where young people’s experiences, ideas, and perspectives are respectfully acknowledged, and well-integrated institutionally for structured engagement. Power-sharing is a key tenet for effective participation. Young people should not only be engaged as beneficiaries and implementers of AMR interventions but should be recognized and engaged as experts and leaders in designing the interventions to best address their context [33]. In the context of AMR, it is important that accountability mechanisms at different engagement levels ensure effective implementation, and evaluation in different contexts where youth are engaged. Strong bidirectional relationships have been shown to exist in interventions where youth are involved in designing of programmes [34]. While acknowledging the diversity of youth, it is important that intentional efforts are taken to ensure youth from underserved and marginalized communities are integrated onto the different AMR interventions as per context [35].

**Fig 1:**
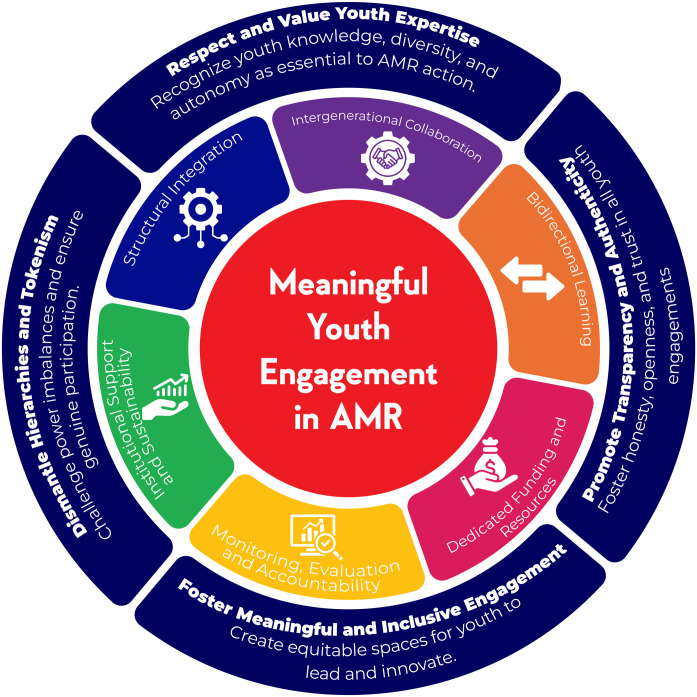
Meaningful Youth Engagement in AMR Wheel.

There are several elements and strategies that should be considered when engaging youth in AMR interventions across the landscape which can offer guidance on how to engage youth meaningfully. The elements and strategies have been illustrated in Fig 1:

### Acknowledge, respect, and value youth knowledge and expertise

Youth have in-depth experiential knowledge, and expertise by nature of being young people [5]. This is further reinforced by their diverse experiences based on their diverse educational backgrounds, and skill-sets. It is important that their expertise is genuinely valued in any AMR interventions. Recognizing stakeholders do not have a complete understanding of the needs and experiences of youth helps encourage bidirectional learning, and active listening. This leads to dismantling of exploitative power structures and knowledge hierarchies that reduces patronization and tokenistic engagement [36,37].

### Recognizing diversity among youth

There exists a wide range of diversity among youth in terms of socioeconomic status, ethnic origin, developmental stage, gender and sexuality, disability, culture, cultures, and life-experiences. Youth also experience different contextual constraints. Recognition of diversity breeds an understanding that different youths have different viewpoints that need to be respected and heard when designing interventions.

### Respecting the autonomy and self-determination of youth

As highlighted, youth have diverse expertise that they can utilize across a diverse breadth of AMR interventions. The role of youth engagement in AMR mitigation should not be prescribed or framed to focus on certain interventions such as awareness. Rather, youth engagement should be viewed from a structural and holistic lens that appreciates the autonomy, and competence of youth, encourages self-determination in the interventions they intended to pursue, recognizes their interests, contextual needs and priorities [38].

### Being transparent, and genuine

It is important that stakeholders are transparent with youth from the outset about the goals, objectives, benefits, expectations, and constraints of any AMR intervention in which they will be engaged. Stakeholders should ensure that they clearly communicate the degree of input, amount of commitment from a flexible lens that encourages feedback from youth. The youth-stakeholder partnerships should be clearly defined and genuine in terms of value accrued by all parties involved. In case of changes, it is important that youth are informed before their enactment. Their feedback should be considered, and they should be informed how the changes are being utilized. Any commitment made should also be fulfilled. Being transparent and genuine ensures there is a climate of trust and understanding, and optimizes active participation of youth.

### Fostering meaningful opportunities, and thriving platforms

Meaningful opportunities for the youth can be enhanced by creating platforms that enable them to demonstrate their strengths. Additionally, the design and implementation of interventions aimed at engaging them should align with their goals. Their input should also be validated by enabling them to share the interventions they have co-created and co-implemented. The platforms where they are engaged should be safe, encourage their contributions and opinions to allow them to feel comfortable in expressing themselves [39]. There should also be authentic respect for the input provided by the youth [40].

### Formally recognizing contributions

In any AMR intervention that youth are engaged in, their input and expertise should be formally recognized. The recognition should align with the resources allocated for the intervention. Meaningful recognition is diverse and may include: remuneration congruent with the input, compensation for time spent, certification, recommendation and references, authorship for publications, recognition in reports, and communicating their engagement during dissemination among other ways [41]. Before deciding on their contributions, it is important to consult them.

### Deconstructing knowledge hierarchy

Diversity brings together different areas of expertise. It is important to deconstruct knowledge hierarchies and acknowledge the knowledge and expertise of all present as significant in the design and implementation of interventions. Knowledge hierarchy occurs when one form of knowledge, especially explicit knowledge gained through formal education and from scientific knowledge, is seen as superior to tacit knowledge gained from experiences [42]. In such practices, some voices are regarded as more important than others, and this diminishes respect for youth voices. It also creates power imbalances leading to decisions being primarily driven by the privileged knowledge with other inputs such as those from youth receiving less consideration [43].

### Promoting clear and transparent communication

Some AMR interventions contain a lot of jargon that may hinder effective communication between youth and other stakeholders. It is important to employ science communication in simplifying jargonized language through effective listening and well-informed feedback, while avoiding oversimplification that could be perceived as diminishing the abilities of the youth [44].

### Promoting authentic feedback strategies to dismantle manipulative steering engagement

Authentic engagement involves intentional listening to feedback, and taking the feedback into account even when it is not what the stakeholders in an intervention were hoping to receive [23]. Where an intervention has an existing agenda, stakeholders should completely avoid steering youth towards the responses they hope to elicit. This is a retrogressive manipulative practice which disregards the entire process of meaningful youth engagement, promotes tokenism and erodes opportunities for meaningful contributions from youth.

### Deconstructing tokenistic and patronizing approaches

Tokenism and patronization are major barriers in meaningful youth engagement. Tokenism in youth engagement can be described as an engagement where youth are engaged “for show or to tick a box” and their inputs are not recognized and taken into consideration in decision-making processes [45,46]. Patronization in this context refers to situations where the youth are treated as “lesser experts” or “being too young to understand” which leads to dismissal of their inputs and undermines their capability. Such practices portray epistemic injustice where their credibility as experts in various areas is unfairly devalued due to their identity as young people. It is important to be aware and intentionally deconstruct the these approaches through the strategies shared above when engaging with youth in AMR interventions [46].

### Strategies for enhancing youth engagement in AMR initiatives

Enhancing youth engagement requires intentional efforts to transform existing structures. It is not enough to merely provide young people with a platform to voice their opinions; true engagement demands giving them the authority, resources, and autonomy to also make decisions. That intergenerational partnership in substantive decision-making has not materialized because youth remain marginalized [47].

Engagement strategies for youth in the fight against antimicrobial resistance (AMR) must go beyond merely building capacity and knowledge. While such preparation is essential for effective advocacy and leadership, it is insufficient without fostering meaningful partnership. Youth must be given real opportunities to apply their skills in decision-making processes, policy creation, and program implementation related to AMR initiatives. Without these opportunities, capacity-building alone becomes counterproductive, leaving young people disillusioned and reinforcing the facade of engagement, ultimately limiting progress in combating AMR. A shift to an equitable and inclusive approach can create measurable progress and foster accountability at local, national, and global levels.

The following strategies outline practical approaches to embedding youth at the center of AMR initiatives:

### Structural integration

The role and vision for youth engagement in AMR initiatives need to be clearly defined based on the principles of collective responsibility and inclusivity. Ensuring that young people are recognized as key stakeholders in global health discourse, rather than passive beneficiaries, is critical for effective integration. Policy frameworks that formally institutionalized youth as partners in the fight against AMR should be incorporated at all levels, ensuring representation in governance structures such as advisory boards and technical teams. This structural inclusion enhances the chances of reflecting the perspectives of younger generations while fostering innovation and sustainability in AMR efforts [48].

Studies have shown that failing to engage young people structurally results in missed opportunities to understand the social determinants influencing individual and community health behaviors [49]. When young people are positioned within decision-making bodies, they contribute fresh perspectives that lead to more responsive and effective policies. For example, global institutions such as the World Health Organization (WHO) and the United Nations (UN) have recognized the value of youth advisory councils in shaping policies and interventions related to public health [50]. Establishing legally binding mandates for youth inclusion in AMR policymaking can ensure sustained participation beyond tokenistic engagement.

### Intergenerational collaboration

For effective AMR interventions, youth must not only be included but actively engaged in dialogue with older technical experts. Creating spaces for intergenerational collaboration allows young people to share their experiences and innovative ideas while benefiting from the expertise of senior professionals. This approach fosters mutual respect, knowledge exchange, and a more comprehensive understanding of AMR challenges, leading to more effective interventions [36].

Mentorship programs that pair young advocates with experienced professionals in AMR-related fields have been proven to accelerate youth capacity-building while fostering intergenerational learning. Studies suggest that structured youth-adult partnerships enhance leadership development and contribute to more equitable decision-making processes [45]. For instance, the International Union for Conservation of Nature (IUCN) has successfully integrated intergenerational dialogue into its sustainability programs, proving that collaborative governance leads to improved policy outcomes [48].

Moreover, collaborative research initiatives that bring together youth and senior scientists can advance the development of innovative AMR solutions. Programs that enable youth to co-author policy briefs and scientific publications with senior experts not only enhance their credibility but also ensure that AMR strategies remain dynamic and inclusive [51]. Establishing structured mentorship, co-leadership programs, and cross-generational research initiatives can help bridge the knowledge gap while reinforcing the shared responsibility of addressing AMR challenges.

### Bidirectional learning

Building youth capacity in the context of AMR is crucial, but it must go beyond theoretical training and be accompanied by real-world opportunities for participation. While technical knowledge and advocacy training provide a foundation, young people must be actively involved in shaping and implementing AMR-related policies. Research shows that youth engagement in leadership roles enhances their ability to contribute meaningfully to public health initiatives and policy development [52].

Structured leadership programs, internships, and fellowships within AMR-related organizations are essential mechanisms for ensuring that young people gain hands-on experience. Studies have demonstrated that when young professionals are given opportunities to lead AMR-related campaigns and community interventions, they develop stronger competencies in advocacy, policy analysis, and project implementation. For example, the Global Health Corps Fellowship has successfully integrated youth into health policy work, proving that experiential learning is key to sustaining engagement [53].

Additionally, co-designing AMR curricula with young professionals ensures that training programs remain relevant to the evolving challenges of antimicrobial resistance. A participatory approach to education and skills development ensures that youth gain practical experience while maintaining ownership of the solutions they advocate for. The most effective knowledge exchange includes opportunities for dialogue, action planning, and real-world implementation of ideas, which solidify youth leadership in AMR initiatives.

### Dedicated funding and resources

One major barrier to effective youth participation in AMR initiatives is the lack of access to financial and logistical support. Studies indicate that youth-led initiatives often struggle to secure the necessary funding, limiting their ability to implement sustainable AMR projects [54]. Data on the percentage of AMR funding specifically allocated to youth programs remains scarce, highlighting the need for transparent and targeted investment in youth-led efforts.

To address this gap, AMR-focused funding mechanisms must be established to support small-scale, youth-led programs in communities affected by antimicrobial resistance. Research has demonstrated that when young people have direct access to grants and funding opportunities, they are more likely to develop innovative, context-specific solutions that drive policy and behavior change [47]. The creation of dedicated youth funding streams within major global health initiatives would ensure that young advocates have the financial backing needed to sustain their work.

Furthermore, facilitating partnerships between youth-led organizations, international agencies, governments, and the private sector can enhance financial and technical support. Studies have shown that cross-sector collaborations improve the effectiveness and sustainability of youth-driven health initiatives [50]. Establishing co-funding mechanisms and incentivizing corporate sponsorships for youth AMR programs can further enhance the capacity of young people to contribute meaningfully to AMR interventions.

Finally, incorporating youth-specific AMR grant programs within existing research and development funding schemes can promote inclusivity. By prioritizing youth-led innovation and entrepreneurship in AMR strategies, global health organizations can unlock fresh perspectives and solutions that would otherwise be overlooked.

### Monitoring, evaluation, and accountability

To ensure meaningful and sustained youth engagement, it is essential to establish mechanisms for monitoring and evaluating youth contributions to AMR initiatives. This could include periodic youth-led assessments of AMR programs and policies, where young people actively participate in tracking progress and suggesting improvements. Accountability frameworks that involve youth in evaluation processes will not only enhance the legitimacy of AMR efforts but also ensure that young people remain empowered in shaping the direction of AMR strategies [49].

Incorporating youth representation in AMR oversight committees and advisory bodies can further institutionalize their role in holding stakeholders accountable. By actively participating in governance structures, young people can provide continuous feedback on policies and interventions, ensuring that their perspectives remain central to decision-making [50]. Additionally, the establishment of independent youth accountability networks can create a structured approach for monitoring AMR commitments. These networks, led by young professionals and advocates, can serve as watchdogs to track government and institutional pledges on AMR, ensuring transparency and sustained action. Public reporting mechanisms, such as youth-driven scorecards and shadow reports, can also be leveraged to assess the effectiveness of AMR interventions and advocate for policy adjustments when necessary.

### Operational framework

We have developed the operational framework in Table 1 to offer key actionable steps that governments, civil society organization, donors and other key stakeholders can take up to advance meaningful youth engagement within their AMR interventions. The framework provides guidance for operationalizing the strategies highlighted in the section above.

**Table 1:** Operationalizing Meaningful Youth Engagement in AMR.

Category	Objectives	Practical Steps	Documented Examples in Literature
Structural Integration	Embed youth as formal, influential stakeholders in AMR policy, governance, and program design.	Identify entry points Define roles and terms of reference Appoint youth representatives Build trust and establish shared goals Identify and address power imbalances Review annually	WHO have institutionalized a Youth Advisory Council that inform global health policy, demonstrating improved responsiveness and innovation when youth are structurally integrated [55]
Intergenerational Collaboration	Build mutual trust, mentorship, and learning between youth and senior experts.	Create a structure that supports mutual participation Offer a range of participation methods (in-person workshops, virtual meetings, asynchronous feedback) to accommodate diverse needs. Hold quarterly co-learning sessions Document outcomes	IUCN intergenerational dialogue programs and WHO mentorship models have shown that structured youth–adult partnerships improve policy outcomes [47]
Capacity Building and Skills Development through Active Participation	Strengthen youth technical, leadership, and research capacities through experiential learning.	Identify gaps in youth knowledge through research Provide training or resources to ensure youth can contribute meaningfully, particularly in understanding methods and processes. Implement a buddy system where youth can be paired with an adult mentor for support and mentorship.	The Investment Readiness Program by SDSN Youth is a virtual 4-month accelerator program that provides handholding for youth-led startups in terms of mentorship, funding, and a structured approach to grow their startups [56]
Access to Funding and Resources	Provide equitable and transparent access to financial and logistical support for youth-led AMR initiatives.	Co-design a youth microgrant scheme Create youth budget line during annual strategic plan meetings Simplify application processes for youth Encourage creative ways of submitting project ideas, such as videos, infographics, etc.	During the World AMR awareness week (WAAW), South Centre provides funding and technical support to both CSOs and youth-led organizations for their AMR advocacy projects [57].
Monitoring and Accountability	Ensure that youth are active agents in evaluating AMR progress and shaping future strategies.	Develop process and outcome indicators Conduct routine data collection through check-ins and a review process. Hold debriefing meetings for troubleshooting Create channels for continuous feedback Acknowledge and highlight youth inputs during presentations or in policy briefs.	Youth-led accountability networks under the SDG Accountability Project by Restless Development demonstrate how young people can drive evaluation and transparency [50].

## Conclusion

Sub-Saharan Africa bears the highest burden of AMR, coupled with limited mitigation interventions [9,58]. This dire situation calls for urgent and accelerated investment in AMR mitigation interventions among all stakeholders. Meaningful youth engagement is not only a moral imperative but also well-positioned as a key catalyst in sustaining interventions across Africa. Engaging the youth meaningfully will ensure that AMR remains prioritized in the coming years and that there is a large pool of multidisciplinary professionals and active citizens dedicated to mitigating AMR. To achieve this engagement, there must be intentionality from all stakeholders involved; ensuring that youth expertise and contributions are adequately recognized, there is dedicated funding support for their interventions, and that their autonomy is respected. Additionally, their inclusion in decision-making structures must be institutionalized at the country, regional, and international levels. This will create an environment that allows for intergenerational collaboration based on trust and integrity, and promotes active bidirectional capacity strengthening. These actions will trigger diverse, innovative, and locally-owned AMR interventions within Africa.
